# Functionalization of microparticles with mineral coatings enhances non-viral transfection of primary human cells

**DOI:** 10.1038/s41598-017-14153-x

**Published:** 2017-10-27

**Authors:** Andrew S. Khalil, Xiaohua Yu, Angela W. Xie, Gianluca Fontana, Jennifer M. Umhoefer, Hunter J. Johnson, Tracy A. Hookway, Todd C. McDevitt, William L. Murphy

**Affiliations:** 1 Department of Biomedical Engineering-University of Wisconsin-Madison, Madison, WI USA; 2 Department of Orthopedics and Rehabilitation-University of Wisconsin-Madison, Madison, WI USA; 3 The Materials Science Program-University of Wisconsin-Madison, Madison, WI USA; 4 The Stem Cell and Regenerative Medicine Center-University of Wisconsin-Madison, Madison, WI USA; 5 Department of Bioengineering & Therapeutic Sciences-University of California, San Francisco, San Francisco, CA USA; 6 Roddenberry Center for Stem Cell Biology & Medicine-Gladstone Institutes, San Francisco, CA USA

## Abstract

Gene delivery to primary human cells is a technology of critical interest to both life science research and therapeutic applications. However, poor efficiencies in gene transfer and undesirable safety profiles remain key limitations in advancing this technology. Here, we describe a materials-based approach whereby application of a bioresorbable mineral coating improves microparticle-based transfection of plasmid DNA lipoplexes in several primary human cell types. In the presence of these mineral-coated microparticles (MCMs), we observed up to 4-fold increases in transfection efficiency with simultaneous reductions in cytotoxicity. We identified mechanisms by which MCMs improve transfection, as well as coating compositions that improve transfection in three-dimensional cell constructs. The approach afforded efficient transfection in primary human fibroblasts as well as mesenchymal and embryonic stem cells for both two- and three-dimensional transfection strategies. This MCM-based transfection is an advancement in gene delivery technology, as it represents a non-viral approach that enables highly efficient, localized transfection and allows for transfection of three-dimensional cell constructs.

## Introduction

Advancements in gene delivery technology are of great interest for both clinical and basic biomedical research applications^1–4^. Gene delivery strategies are broadly classified as non-viral or viral delivery methods^4,5^. Viral gene delivery approaches have high gene transfer efficiencies but limited capsid carrying capacity, and safety concerns about viral capsid immunogenicity as well as insertional mutagenesis limit their therapeutic translation^5–7^. Non-viral delivery approaches can be further subdivided into physical and chemical methods^5^. Physical methods include the use of ballistics^8^, electric fields^9^, osmotic pressure, or physical injection^10^ to disrupt the cell membrane and deliver nucleic acids directly to the cytoplasm^5^. Some of these physical methods have been refined to achieve high efficiencies relative to viral delivery with low toxicity *in vitro*, but have limited clinical promise in large animals or humans^5^.

Chemical transfection methods utilize reagents such as cationic lipids, polymers/dendrimers, and peptides to condense nucleic acids into complexes that can be endocytosed, primarily through clathrin- or caveolae-mediated mechanisms^11–14^. Typical transfection reagents serve two primary purposes: 1) to condense the nucleic acids to a size that is compatible with endocytosis, and 2) to mitigate electrostatic repulsion between the negatively charged nucleic acid phosphodiester backbone and the cell surface^5,11,13,15,16^. Some reagents also disrupt endosomal membranes to facilitate entry of nucleic acids into the cytoplasm^7,13,17^. However, chemical methods generally yield lower transfection efficiencies than physical or viral gene delivery methods and are often cytotoxic^5^. In addition, chemical methods typically cannot achieve high transfection efficiencies in three-dimensional (3-D) cell culture or *in vivo* due to additional challenges such as changes in cellular uptake of lipoplexes^18^ and physical barriers preventing access to the interior cells of 3-D constructs or tissues^19^. Thus, there is a need to improve the efficiency of chemical transfection methods, for both therapeutic and research applications.

Our group previously demonstrated that the application of biomimetic mineral coatings on cell culture substrates can enhance non-viral transfection of primary human cells^20,21^. Upon incubation of microparticles in a simulated body fluid containing the ion species and concentrations of human blood plasma, modified to contain 2X calcium (mSBF), a mineral coating forms on the microparticle surface via a nucleation and growth mechanism. These coatings are biocompatible, bioresorbable, charged, and have a high degree of nanometer-scale porosity, allowing for efficient delivery for a range of different biomolecules^20,22–26^ including DNA complexes for chemical transfection. The coating properties, such as nanotopography and dissolution rate can be fine-tuned through modifications to the mSBF composition^24^, including changes in the concentrations of ionic calcium, phosphate, carbonate, and other inorganic dopants (S1), all of which may influence the coating’s capacity to bind and deliver DNA complexes^20,25,27,28^.

Previous studies have explored the use of microparticles to improve chemical transfection by increasing the extent of interactions between nucleic acid complexes and the cell surface^29,30^. Here, we demonstrate that functionalization of microparticles with mineral coatings further enhances their capacity to transfect cells. Specifically, we hypothesized that these mineral coatings would improve the microparticles’ capacity to bind soluble lipoplexes out of solutions^29,30^. Additionally, we hypothesized that the microparticle format would enable higher transfection efficiency to be achieved in 3-D, via incorporation of mineral-coated microparticles (MCMs) throughout 3-D cell constructs. MCMs reduced cytotoxic effects commonly associated with chemical transfection reagents, and improved transfection efficiency for several primary human cell types including dermal fibroblasts (hDF), embryonic stem cells (hESC), and mesenchymal stromal cells (hMSC). In addition, we showed that improved transfection can be achieved with a variety of microparticle core materials, and demonstrated efficient localized transfection via MCMs in both two-dimensional (2-D) and 3-D cell culture formats.

## Results

### Incubation of microparticles in specified mSBF solutions resulted in mineral coatings with distinct nano-structure and stability characteristics

**Figure 1:**
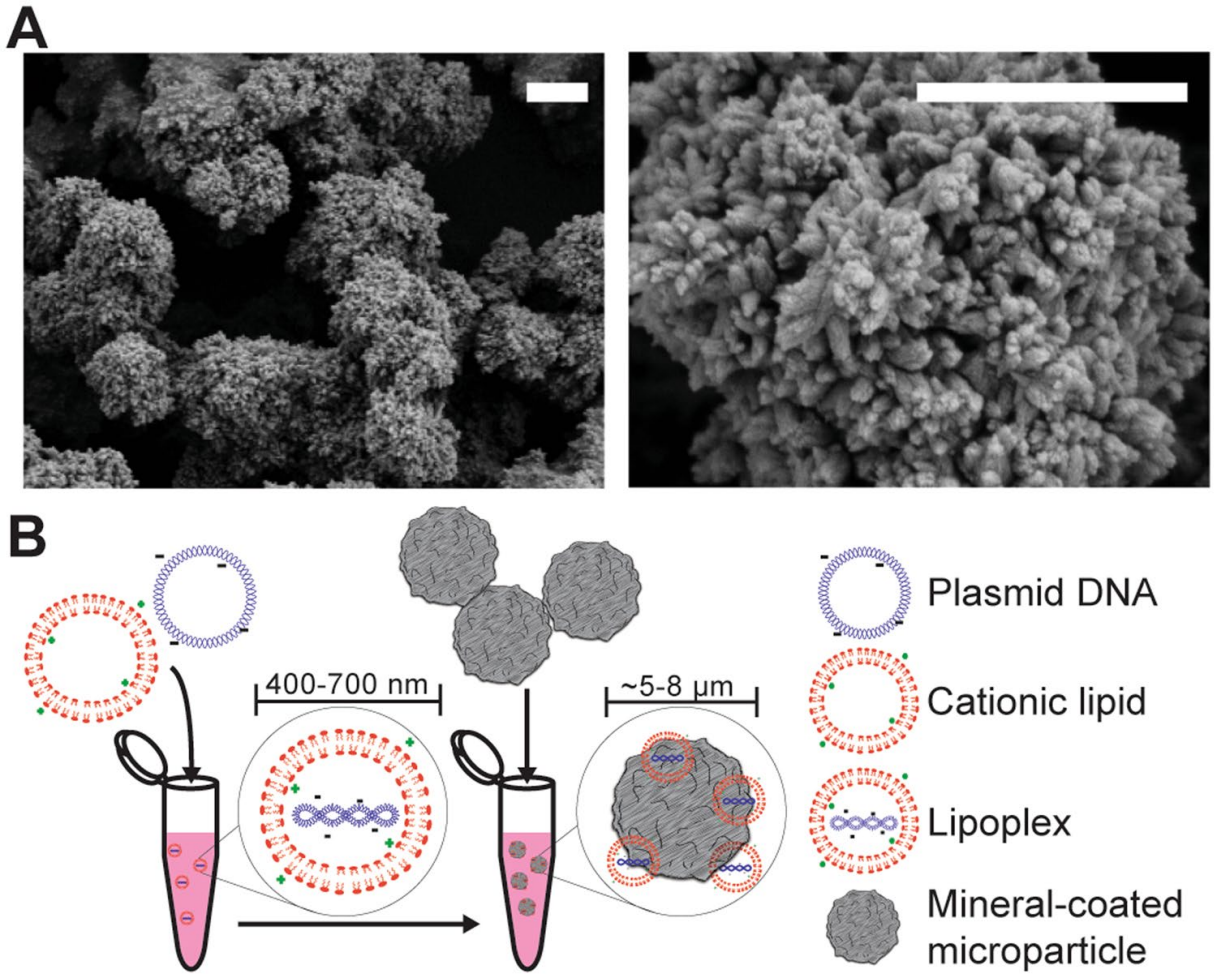
Mineral-coated microparticles (MCMs) for non-viral transfection, formed in 4.2 mM NaHCO_3_ + 100 mM NaF-containing mSBF. (**A**) Scanning electron micrograph of MCMs (left), which are ~5–8 μm in diameter. A single MCM (right), presenting a nanostructured coating. (**B**) Schematic for loading MCMs with pDNA-lipoplexes. Scale bars = 2 µm.

Hydroxyapatite powder incubated in mSBF for 5 days yielded MCMs between 5–8 µm in diameter with calcium phosphate coatings (Fig. 1A). The specific mSBF formulation (S1) dictated coating properties, such as the coating stability and nanometer-scale morphology (S2A). Specifically, increasing mSBF carbonate concentration increased MCM dissolution rate, as measured by an increase in 3-day cumulative calcium release from 221.9 ± 21.2 nmol Ca^2+^/mg MCMs (4.2 mM carbonate) to 291.9 ± 15.8 nmol Ca^2+^/mg MCMs (100 mM carbonate) (S2A right). The inclusion of sodium fluoride in the coating solution correlated with a 2.4-fold decrease in 3-day cumulative calcium release for 4.2 mM carbonate MCMs but had no effect on calcium release from 100 mM carbonate MCMs (S2A right). In addition, fluoride inclusion resulted in a change in nano-scale morphology from a “plate-like” to a “needle-like” structure (S2A left, middle). Incubation of MCMs with soluble lipoplexes (Fig. 1B) resulted in binding efficiencies of 54.0 ± 2.6% and 67.6 ± 3.7% after 30 minutes and 2 hours, respectively (S2C).

### MCMs improved non-viral transfection of primary human dermal fibroblasts (hDFs) in a two-dimensional (2-D) cell culture format

**Figure 2:**
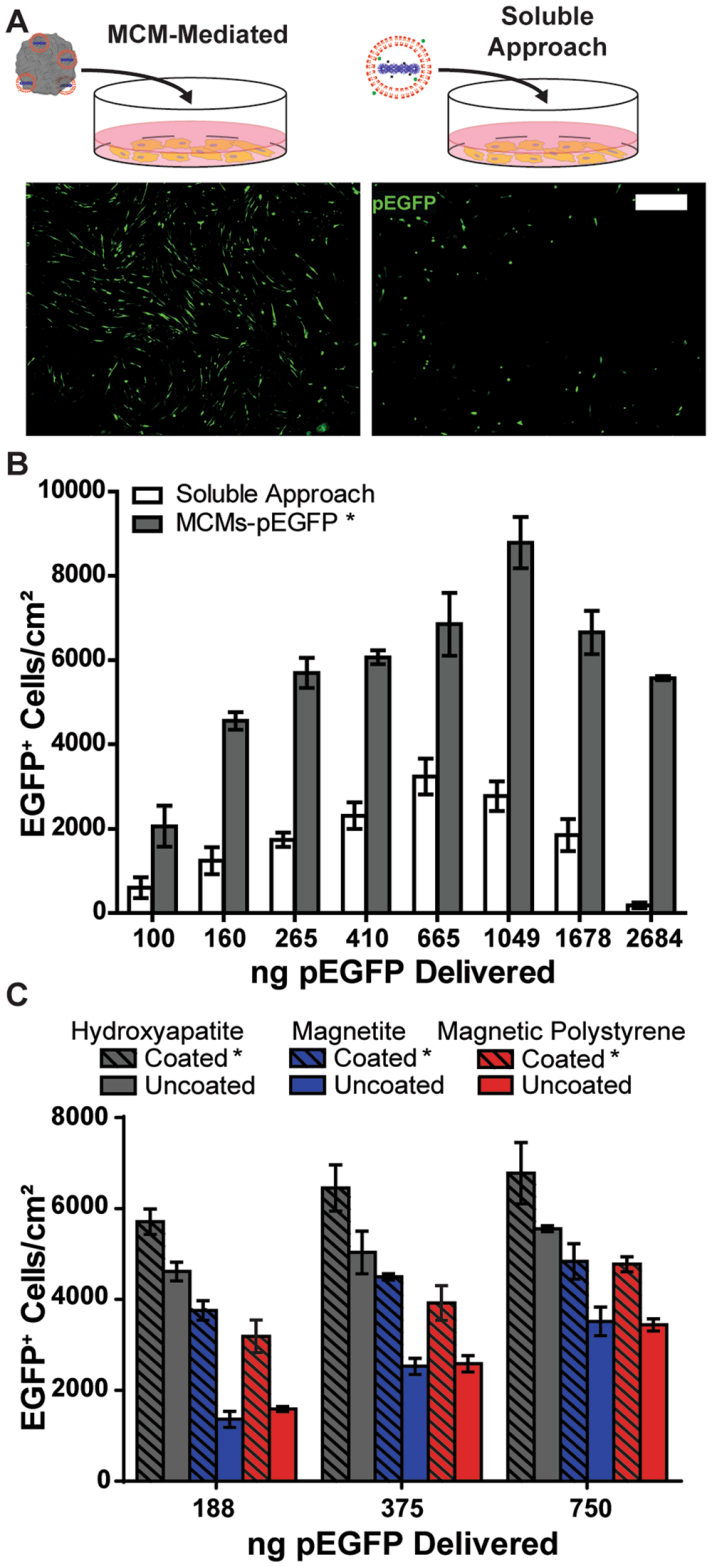
Comparison between soluble and MCM-mediated delivery of pEGFP-lipoplexes to human dermal fibroblasts (hDFs). (**A**) Schematics and representative fluorescent micrographs from soluble and MCM-mediated delivery strategies. Cells were seeded at ~7500 cells per cm^2^, transfected after 24 hrs, and transfection efficiency quantified at 36 hrs post-transfection. (**B**) Comparison of the resulting number of EGFP+ cells per cm^2^ between soluble and MCM-mediated delivery. *2-way ANOVA p-value < 0.001 (**C**) Mineral coating (4.2 mM NaHCO_3_ + 100 mM NaF) enhanced the transfection capacity of hydroxyapatite, magnetite, and magnetic polystyrene microparticles. Scale bar = 100 µm. *2-way ANOVA p-value < 0.005.

Compared to a standard soluble lipoplex delivery approach (“soluble approach”), the MCM-mediated transfection resulted in a 4-fold increase in EGFP+ cells/cm^2^ (Fig. 2A,B). MCMs promoted an increase in transfection efficiency irrespective of the underlying core material used (Fig. 2C), indicating that the mineral coating is critical to the observed increase. Consistent with previous studies describing microparticles that bind lipoplexes out of solution^29,30^, we observed that uncoated hydroxyapatite (HA), magnetic tissue culture polystyrene (TCPS), and magnetite (Fe_3_O_4_) microparticles bound soluble lipoplexes with ~70% efficiency (S2D). Mineral coatings on HA, TCPS, and magnetite microparticles further increased the transfection efficiency of these materials 1.2-, 2.0-, and 1.6-fold, respectively, over uncoated microparticles. Notably, this increase in transfection efficiency did not appear to be due to changes in lipoplex binding capacity, as there was no statistically significant difference in lipoplex binding between MCMs and uncoated microparticles (S2D). We also observed no dependence on particle size when transfecting with 2, 6, and 16 µm MCMs (S3).

### MCM-mediated transfection decreased cationic lipid-associated cytotoxicity

**Figure 3:**
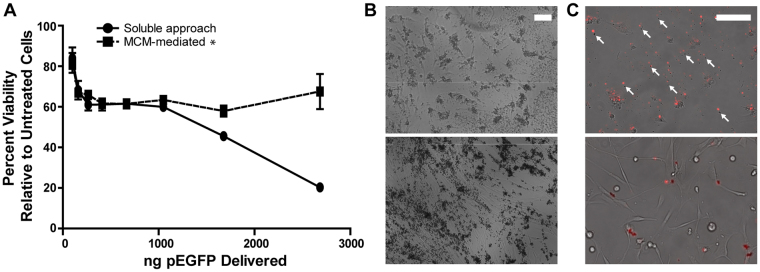
MCMs formed in 4.2 mM NaHCO_3_ + 100 mM NaF-containing mSBF reduce cytotoxicity of cationic lipids during transfection of hDFs. (**A**) Viability comparison between soluble and MCM-mediated delivery using a resazurin reduction viability assay. (**B**) Representative micrographs 24 hours after hDF transfection. MCM-mediated transfection (bottom) results in spread cell morphology while soluble transfection (top) results in rounded cells. (**C**) We observed visible lipoplex agglomerates (arrows) 24 hours after addition of soluble lipoplexes (top) which coincided with increased cell debris. No agglomerates were observed with MCM-delivered lipoplexes (bottom) and labeled pDNA was restricted to the MCMs. This absence of agglomerates with MCM-mediated delivery coincided with less cell debris observed relative to the soluble lipoplex delivery method. Scale bars = 100 µm. *2-way ANOVA p-value < 0.0001.

We used a resazurin conversion assay to measure differences in viability of hDFs transfected via soluble or MCM-mediated delivery, over a range of lipoplex concentrations. At the highest pDNA concentration tested, the soluble delivery approach resulted in only 20.3% cellular viability relative to untreated controls. This result was further supported by the observation of hDFs with rounded morphology, indicative of poor cell health, in conditions transfected by soluble delivery (Fig. 3A,B). In contrast, MCM-mediated delivery of an equivalent concentration of pDNA maintained 67.5% cell viability relative to untreated controls (Fig. 3A), and hDFs transfected via MCMs exhibited a spread morphology that is typical for this cell type in culture. This reduction in cytotoxicity via MCMs, however, coincided with a reduction in transfection efficiency above 1049 ng pDNA/well (Figs 2B and 3A). Using rhodamine-labeled pEGFP, we observed that in conditions transfected via the soluble approach, cytotoxic lipoplex agglomerates formed outside the cells within 7 hours after lipoplex addition. In contrast, no visible agglomerates formed via the MCM-mediated approach, and we observed labeled lipoplexes only within cells or on the MCM surface (Fig. 3C). Lastly, we evaluated transfection via the MCM-mediated method using additional commercially available transfection reagents, including another cationic lipid-based reagent (LyoVec™) and several polyethylenimine (PEI)-based cationic polymers (S5). For each of the reagents tested, MCM-mediated delivery resulted in significant increases in transfection efficiency at high pDNA concentrations, compared to those achieved by the soluble delivery approach. The MCMs reduced the cytotoxicity of the cationic lipid reagent and protamine-branched PEI combination relative to soluble approaches, but not of the cationic polymers without protamine; however, we observed no transfection in the soluble conditions for either linear PEI or branched PEI alone.

### Transfection from nucleic acid-laden MCMs required localized cell-MCM interactions

**Figure 4:**
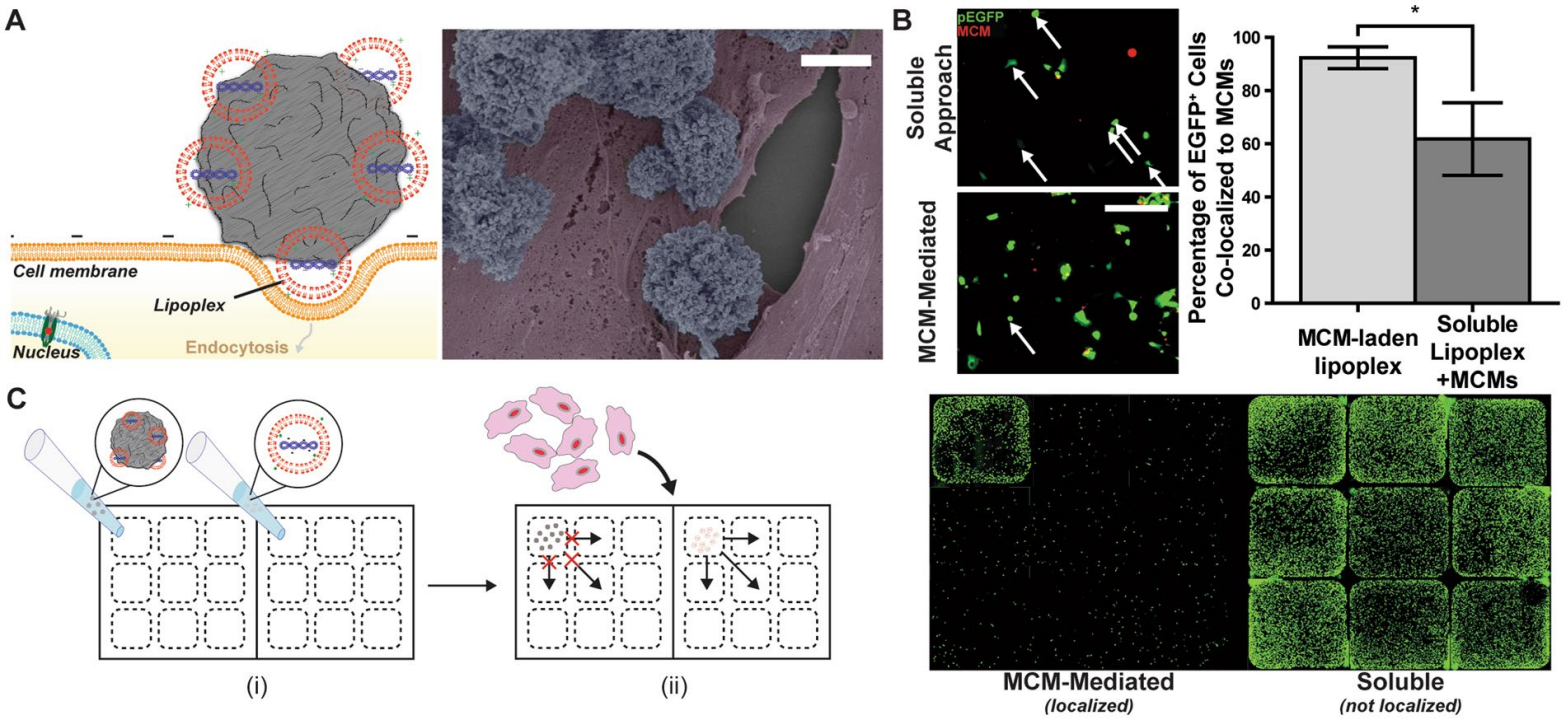
MCMs facilitate non-viral transfection via a localized interaction at the MCM-cell interface. (**A**) (left) Schematic for MCM-mediated delivery at the MCM-cell interface. (right) Scanning electron micrograph of MCMs interacting with hDF (pseudo colored). The particles are not taken up by the cell, but rather bind to the cell and deliver lipoplexes locally. (**B**) (left) MCM-mediated transfection of human embryonic kidney cells (HEK293) versus soluble lipoplex transfection in the presence of rhodamine-labeled MCMs. With MCM-mediated transfection, greater than 90% of EGFP+ cells colocalized with MCMs, while the soluble approach resulted in many EGFP+ cells that were not associated with a microparticle. Arrows represent EGFP+ cells not associated with a MCM. Scale bar = 50 µm (right) Quantification of percent colocalization of EGFP+ cells with MCMs. (**C**) Localized transfection within a mixed-media culture dish. (i) EGFP-lipoplex-laden MCMs (left) and soluble lipoplexes (right) were pipetted into the upper-left microwell and allowed to settle. (ii) After 10 min, HEK293s were seeded into the mixed-media macrowells. EGFP + HEK293s were observed primarily in the upper-left microwell for the MCM-mediated delivery, but were found uniformly throughout the macrowell for the soluble lipoplex delivery. *p-value < 0.05.

Scanning electron micrographs of hDFs cultured with MCMs showed the microparticles interacting at the cell membrane rather than being internalized (Fig. 4A), suggesting that MCM-mediated transfection occurs via delivery of lipoplexes to the cell membrane for subsequent cellular uptake. To test the requirement of localized cell-MCM interactions for successful MCM-mediated transfection, we used rhodamine-labeled microparticles to compare MCM-mediated delivery to a scheme in which soluble EGFP lipoplexes were delivered in combination with (but not bound to) rhodamine-labeled MCMs. With the MCM-mediated delivery method, greater than 90% of EGFP+ HEK293s colocalized with rhodamine-MCMs. In contrast, we observed only 60% colocalization of EGFP+ cells with rhodamine-MCMs in the soluble lipoplex delivery method (Fig. 4B), which was not statistically different from the 48% colocalization of total (DAPI+) cells with MCMs (S6).

### MCMs increased the rate and extent of lipoplex delivery to the cell membrane

Lipoplexes bound to MCMs settled to the bottom of the culture well faster than soluble lipoplexes, as shown with time-lapse microscopy of rhodamine-labeled pDNA lipoplex delivery (S7). In addition, time-lapse microscopy of MCM-cell co-cultures demonstrated an affinity of MCMs for the cell membrane (S8A). Rhodamine-labeled MCMs demonstrated a 9.5-fold increase in Pearson colocalization coefficient with Cell Tracker Green-labeled hDFs (S8B) from 0 to 12 hours after MCM addition. We next evaluated localized transfection in a multi-well plate format. Using a multiwell co-culture device that physically separated nine cell culture microwells but allowed for sharing of media between the microwells, we found that lipoplex-laden MCMs rapidly settled and localized transfection to a single MCM-containing well. Conversely, soluble lipoplexes delivered to a single well diffused outward into the shared media and transfected cells throughout the co-culture device (Fig. 4C). Lastly, we observed that cells cultured in direct contact with MCMs produce more endosomes than cells cultured alone, as shown by up to an 18-fold, dose-dependent increase in the number of pHrodo Green-Dextran+ endosomes (S9A). In addition, cells cultured with MCMs appeared to have a greater incidence of cytoplasmic release of the pHrodo Green-Dextran beads (S9B).

### MCMs improved reverse transfection of H1 human embryonic stem cells (hESCs)

**Figure 5:**
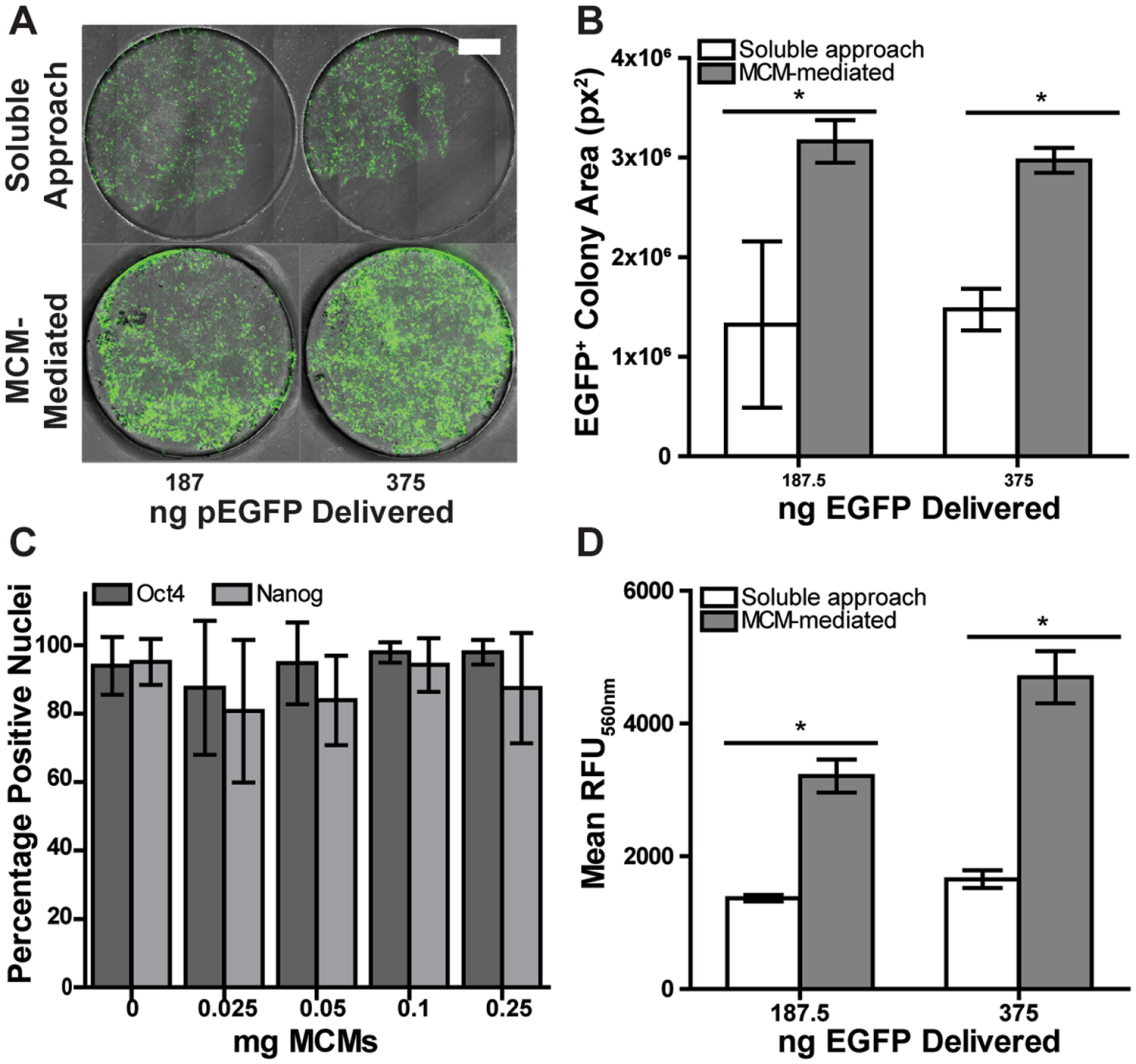
MCMs improve reverse transfection of hESCs. (**A**) Merged FITC and phase-contrast micrographs of reverse transfection in human embryonic stem cells (H1 hESCs) in microwells. (**B**) Quantification of percent EGFP+ area of reverse-transfected H1 hESC colonies. (**C**) Influence of MCMs on pluripotency markers Oct4 and Nanog after 48 hrs of culture. (**D**) Mean EGFP expression of EGFP+ colony fraction, as measured by fluorescence intensity. Scale bar = 500 µm. *p-value < 0.05.

Reverse transfection is a technique whereby nucleic acid delivery takes place from beneath the cell layer or in a cell suspension prior to cell attachment to the culture substrate. Previous studies have demonstrated that this approach is a more effective strategy than traditional transfection for several difficult-to-transfect cell types^21,23,31^, including hESCs. We compared MCM-mediated transfection to soluble lipoplex transfection via placement of soluble or MCM-bound lipoplexes in Matrigel-coated polydimethylsiloxane (PDMS) microwells prior to hESC seeding. Lipoplex-laden MCMs rapidly adsorbed to the Matrigel coating within 10 minutes (S10A,B). hESCs were reverse-transfected via MCMs, resulting in a 2.8-fold increase in EGFP+ colony fraction, and a 3.4-fold increase in mean green fluorescence intensity of the EGFP+ colony fraction (Fig. 5A–D) compared to the reverse transfection efficiency achieved with soluble lipoplexes. In addition, MCM-mediated delivery resulted in a 1.5-fold increase in total cell confluence (Fig. 5A), indicating improved cell retention after transfection as well as a greater overall yield of positively transfected cells. Lastly, we observed no significant differences in the percentage of Oct4+ or Nanog+ nuclei after 48 hours of hESCs cultured directly with MCMs (Fig. 5C), suggesting that the presence of MCMs alone does not result in the loss of these pluripotency-associated transcription factors within a timeframe relevant for transfection.

### MCMs enabled efficient 3-D transfection in cell aggregates

**Figure 6:**
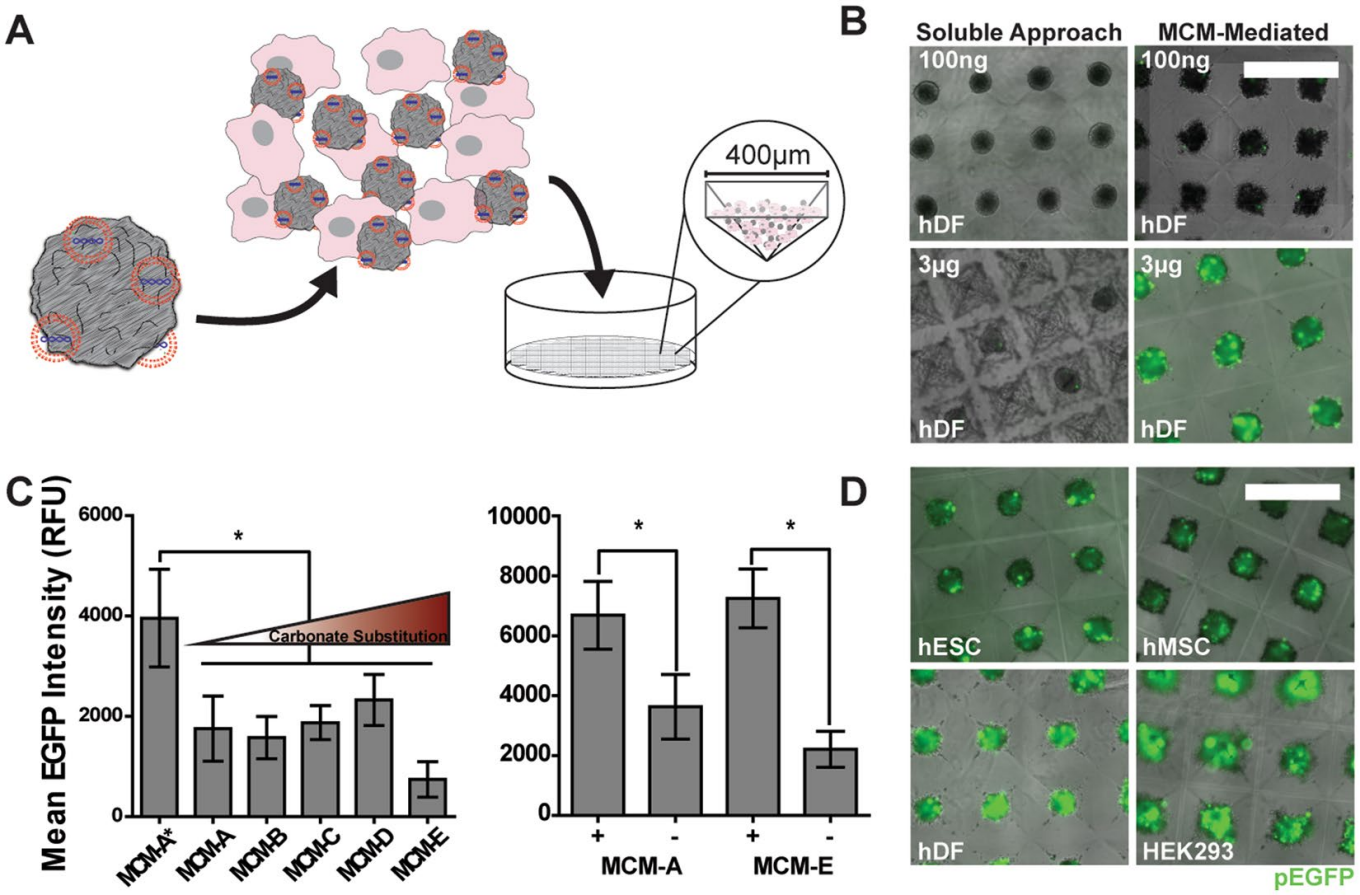
MCMs facilitate efficient 3-D transfection of human primary cells. (**A**) Schematic of MCM incorporation into a cell aggregate interior during forced aggregation. (**B**) Increasing lipoplex concentrations results in cell death for soluble lipoplex delivery, but high levels of transfection for MCM-mediated delivery. (**C**) (left) Enhancement of EGFP expression in transfected hDF aggregates via different mineral coating formulations described in S1. (right) Comparison of EGFP expression in coatings with equivalent carbonate composition, with or without fluoride ions. (**D**) MCM-mediated delivery enables efficient transfection in aggregates of hESCs, hMSCs, hDFs, and HEK293s. Scale bar = 500 µm. *p-value < 0.05.

Three dimensionality in cell culture introduces physical barriers of cell-cell junctions and extracellular matrix, which typically prevent lipoplex access to the interior cells of a 3-D construct or tissue^19^. Here, we utilized cell aggregates formed via centrifugation into agarose microwells (Fig. 6A) as a 3-D cell construct model to compare the efficacy of MCM-mediated delivery versus soluble lipoplex delivery. Soluble lipoplexes were not effective in 3-D transfection, as expected. Increases in soluble lipoplex concentration from 100 ng to 3000 ng pDNA/well did not increase transfection efficiency in 3-D cell aggregates. Notably, at higher soluble lipoplex concentrations, aggregates failed to form and only cellular debris remained in the microwells (Fig. 6B). To overcome the 3-D physical barrier preventing efficient transfection, we next mixed lipoplex-laden MCMs with the singularized cells prior to cell aggregation and formed cell aggregates with MCMs distributed throughout. Via this approach, we observed well-formed aggregates exhibiting uniform EGFP+ expression throughout, as shown by confocal optical sectioning (S11), and minimal cellular debris even at a high concentration of 3000 ng pDNA/well (Fig. 6B). MCM-mediated 3-D transfection was achieved in multiple cell types, including hDFs, HEK293s, human mesenchymal stem cells (hMSCs), and hESCs (Fig. 6D).

### Fluoride-containing mineral coatings significantly increased transgene expression in 3-D

We screened various mineral coating compositions utilizing a 96-well agarose microwell format. We tested coating compositions generated in mSBF containing 4.2, 25, 50, 75, and 100 mM sodium bicarbonate as well as with 4.2 and 100 mM sodium bicarbonate with the addition of 1 mM sodium fluoride. The addition of fluoride to 4.2 and 100 mM sodium bicarbonate MCMs resulted in a 1.8- and 3.3-fold increase in transgene expression, respectively (Fig. 6C). The increase in transgene expression with fluoride-doped mineral coating formulations was not due to differences in lipoplex dosage, as we did not observe a significant difference in lipoplex binding capacity between fluoride-containing and fluoride-free coatings (S2E). However, fluoride-containing mSBF formulations resulted in a transition of the nanometer-scale coating morphology from “plate-like” to “needle-like” (S2A). The addition of fluoride to the 4.2 mM sodium bicarbonate formulations also reduced the coating dissolution rate as shown by 3.5-fold reduction in cumulative calcium release over 3 days (S2A). Taken together, these data indicate that total ion release, nanometer-scale topography, and the presence of fluoride ions at the MCM-cell interface may each influence the mechanism by which MCMs transfer lipoplexes to the cell membrane, ultimately dictating the mineral coating’s capacity to increase microparticle-mediated transfection efficiency.

## Discussion

MCMs improved non-viral transfection of pDNA over a standard soluble lipoplex delivery approach in 2-D culture of human primary cells. We demonstrated that MCMs bound lipoplexes with high efficiency and delivered them to the cell surface, without cell internalization of the MCMs (Fig. 4B). We observed an increase in transfection efficiency of the MCM-mediated delivery method relative to soluble delivery at all lipoplex concentrations examined (Fig. 2B), both below and above the manufacturer’s concentration recommendations. Additionally, we observed a decrease in cytotoxicity of Lipofectamine 2000™ in the presence of MCMs, an unexpected effect not described in previous microparticle-based approaches (Fig. 3A,B). However, this increase in transfection efficiency via the MCM method did not persist above 1049 ng per well condition, despite no further increase in cytotoxicity at these concentrations (Figs 2B and 3A). This result suggests that, at higher MCM concentrations, MCMs begin to inhibit or compete with cellular uptake of lipoplexes to limit transfection without further effects on cell viability. Nevertheless, we observed similar improvements in transfection efficiency or reduction in cytotoxicity relative to soluble delivery when we combined the MCM method with several other commercially available polyplex- and lipoplex-forming reagents (S5), a result that supports the broad applicability of this MCM-mediated approach with other common transfection reagents. However, the effects in these cases were variable and less pronounced, likely due to the lack of equal protocol optimization that we performed for Lipofectamine 2000™. In addition, the mineral coating strategy described here is not restricted to microparticle-based delivery approaches, as the nucleation and growth mechanism for generating mineral coatings is adaptable to a wide range of biomaterial substrates including ceramics^32^, polymers^28,33^, and metals^34^.

Increased efficiency observed in MCM-mediated transfection was critically dependent on the mineral coating. Although MCMs and uncoated microparticles exhibited comparable lipoplex binding efficiency (S2D), the MCMs showed greater transfection efficiency for each of the underlying core materials examined (Fig. 2C). These results indicate that the mineral coating plays an active role in transfection process. Interestingly, we found that MCMs increased the production of endosomes in a dose-dependent manner (S9). We posit that the nanometer-scale morphology of the mineral coating and/or the release of soluble mineral ions at the MCM-cell interface^22^ are critical to the observed increases in endosome production and transfection efficiency. Future studies should aim to examine how these and other properties of the mineral coating influence non-viral gene delivery.

We observed that MCMs bound and concentrated soluble lipoplexes (Figs 4C and S2), and showed an apparent affinity for the cell membrane (S8A,B). These results support our hypothesis that MCMs improve microparticle-mediated transfection by increasing the local concentration of lipoplexes at the cell membrane. The reduction in cationic lipid-associated cytotoxicity observed with MCM-mediated transfection is unique to the microparticle-based approach described here, and constitutes another potential mechanism by which MCMs enhance effective transfection efficiency. Previous work has demonstrated that increases in charge ratio and agglomeration of lipoplexes exacerbate cytotoxicity during chemical transfection^35^. We observed a reduction in visible agglomerates in conditions with MCMs (Fig. 3C), suggesting that MCMs mitigate cytotoxicity by either stabilizing lipoplexes, or by sequestering agglomerates and high charge ratio lipoplexes. Taken together, we propose that MCMs deliver high local concentrations of lipoplexes to the cell membrane while reducing the cytotoxic effects of membrane-disrupting transfection reagents.

The MCM binding and local delivery mechanism afforded efficient and simple spatial patterning of transfection *in vitro*. Discrete placement of lipoplex-laden MCMs to a region of interest allowed for localized transfection of the targeted area (Fig. 4C) in HEK293s, a cell type regarded as easy to transfect^36^. As HEK293s are easy to transfect, release and diffusion of lipoplexes would coincide with observation of EGFP+ cells not closely associated with MCMs. The lack of this observation (Fig. 4B) suggests transfer of lipoplexes in close proximity to the cell membrane is critical for transfection via MCMs (Fig. 4C). Combined with the ability to functionalize microparticles of different core materials (e.g., magnetite or magnetic polystyrene), the utility of MCMs for localized transfection may further enable applications such as facile spatial patterning of gene expression *in vitro*, an area in which previous approaches have required specialized viral vectors^37^, surface coatings^38^, or microfluidic devices^39^.

Advancements in gene delivery to human adult and embryonic stem cells is of great interest for therapeutic and disease modeling applications, but low transfection efficiencies for several stem cell types have remained an obstacle^40,41^. Previous studies have demonstrated that reverse transfection strategies can be efficient for stem cell transfections^31,42^, as delivery from beneath the cell layer at the site of cell adhesion or to cells in suspension results in improved endocytosis^43^. Here, we examined whether the lipoplex-binding capacity of MCMs demonstrated here coupled with the previous established protein-binding capacity of MCMs^24,25,27,44,45^ would afford facile and efficient lipoplex delivery from beneath the cell layer (S10). We demonstrated that MCMs are indeed amenable to reverse transfection of hESCs after adsorption to Matrigel-coated substrates. MCM-mediated reverse transfection resulted in larger colonies, greater EGFP+ colony area percentages, and higher levels of EGFP expression than standard reverse transfection, without decreasing the percentage of Oct4+ and Nanog+ hESCs in short-term culture (Fig. 5C). Taken together, use of MCMs for reverse transfection dramatically increased the total number of transfected hESCs as well as levels of transgene expression, indicating that MCMs may be a uniquely useful tool for gene delivery in pluripotent stem cell applications.

Three-dimensionality in cell culture introduces additional barriers to transfection^19^, as large lipoplexes are unable to penetrate into the interior of 3-D constructs. Additionally, previous studies have observed that the mechanism for nucleic acid internalization differs for cells cultured in 2-D vs 3-D, indicating that efficient 2-D transfection strategies may not translate effectively to 3-D cell culture^18^. We demonstrated that in contrast to the soluble lipoplex delivery approach, which caused rampant cytotoxicity at high concentrations of pDNA, incorporation of MCMs enabled formation of viable cell aggregates across a wide range of pDNA concentrations and could significantly improve transgene expression in 3-D transfection of hDFs, hMSCs, and hESCs (Fig. 6D). The transfected cells were distributed throughout the aggregates (S11), demonstrating that incorporated MCMs bypass barriers created by cells in 3-D tissues. Use of these MCMs for transfection of 3-D constructs may be of utility for controlling gene expression in biomanufacturing processes that utilize cell aggregates, such as organoids, tissue-engineered cell constructs, and potentially as an injectable *in vivo* gene carrier.

We observed large increases in transgene expression of cell aggregates transfected via the MCM method when fluoride was included in the mineral coating. The presence of fluoride ions resulted in two differences in the mineral coating that may explain this observation. First, the introduction of fluoride ions altered the coating’s nanometer-scale morphology from plate-like to spiny, needle-like features (S2A). Additionally, the introduction of fluoride ions into the mineral coating reduced the coating dissolution rate and subsequent ion release (S2A). However, changes in mineral coating composition (i.e., changes in carbonate or fluoride concentration) did not alter the capacity of the MCMs to bind lipoplexes (S2E) although we observed a fluoride-dependent increase in transfection efficiency. Previous studies have identified specific relationships between nanotopography^46^, ion concentration^47^, and osmotic pressure^48^ and endocytosis of DNA complexes and other biomolecules. The findings of these previous studies and the result described here further support a hypothesis that nanometer-scale morphology and/or degradation products of the mineral coating actively influence the transfection process. Future work should aim to further characterize localized effects at the cell-MCM interface to understand how mineral coating compositions and topography influence the presentation and uptake of lipoplexes during transfection.

## Conclusion

Advancement of gene delivery technology as a therapeutic and as a research tool is critically dependent on improving both the safety and the efficiency of delivering nucleic acids to primary human cells. Here, we have described a materials-based approach to address both of these criteria. The application of mineral coatings to microparticles resulted in enhanced non-viral transfection of pDNA-lipoplexes in human primary cells in combination with a reduction in cytotoxicity of common transfection reagents. This enhancement occurred via a localized cell-MCM interaction mechanism in which the coating dissolution rate, nanometer-scale topography, and specific ion composition were all relevant material parameters. We demonstrated the utility of this approach in the form of highly efficient reverse transfection of human embryonic stem cells and in the transfection of 3-D cell constructs. In summary, our findings support a novel approach for improving non-viral transfection efficiency in a broad array of cell types, including difficult-to-transfect cell types such as primary human somatic and embryonic stem cells, as well an enabling strategy for 3-D and localized gene delivery.

## Materials and Methods

### Fabrication of MCMs

Hydroxyapatite powder (Plasma Biotal Limited), magnetite powder (Fe_3_O_4_) (Sigma-Aldrich), magnetite-doped polystyrene beads (Spherotech), or carboxyl-coated polystyrene beads of 2, 6, and 16 µm diameter (Spherotech) were used as microparticle core materials. The core materials were suspended at concentrations of 1, 10, and 0.1 mg/mL, respectively, in mSBF formulated as shown in S1. The suspensions were rotated at 37 °C for 24 hrs, at which point the microparticles were centrifuged at 2,000 g for 2 min, and the supernatant decanted and replaced with freshly made mSBF. We repeated this process daily for 5 days, at which point the MCMs were washed three times with 50 mL deionized water, filtered through a 40 µm pore cell strainer, suspended in 15 mL distilled water, frozen in liquid nitrogen, and lyophilized for 48 hrs. The different core material concentrations were chosen based on maintaining 1 × 10^8^ particles per 50 mL of mSBF. The lyophilized MCMs were then analyzed for nanotopography and calcium release (S2A) as previously described^21,24^.

### Binding capacity of plasmid DNA complexes by MCMs

Enhanced green fluorescent protein-encoding plasmid DNA (pEGFP-N1 from Clontech) was amplified according to manufacturer’s protocols in chemically competent DH5α *E. coli* (Invitrogen), purified using EndoFree Plasmid Purification Kit (Qiagen), and measured for concentration and purity via 260/280 nm absorbance (NanoDrop). The pEGFP-N1 was then tagged following the manufacturer’s protocol with a fluorescent rhodamine dye using the Label-IT Nucleic Acid Labeling Kit (Mirus). Lipofectamine 2000™ (Life Technologies) was added to pEGFP-N1-Rhodamine at a ratio of 2:1 in OptiMEM media (Life Technologies) at a concentration of 30 µg/mL pEGFP-N1. The mixture was incubated at room temperature (RT) for 20 min to allow pDNA complexes to form. The complexes were then combined with MCMs at a mass ratio of 13.33:1 pEGFP-N1-Rhodamine:MCMs and incubated at room temperature on a rotator. The incubation was sampled at 30, 60, and 90 min for the MCM-pEGFP-N1-Rhodamine complex mixture to determine the biding efficiency via loss of fluorescence. To measure pDNA binding at different time points, the sampled mixture was centrifuged briefly to pellet the MCMs and the supernatant collected for fluorometric detection at 510 nm/612 nm excitation/emission of pEGFP-N1-Rhodamine (Fluoroskan Ascent FL). The binding efficiency was measured as loss of fluorescence relative to the fluorescence of the initial solution (S2B,C).

### Comparison of 2-D transfection efficiency

Primary hDFs (ATCC) were cultured in Dulbecco’s minimal essential media (DMEM) with 10 v/v% fetal bovine serum (FBS) and 1000 U/mL penicillin and streptomycin (P/S). MCMs were sterilized via UV-exposure (254 nm for 30 min) and suspended in Opti-MEM (Gibco) at 1 mg/mL. Cells were trypsinized (0.05 wt% trypsin/0.1 mM EDTA) for 5 min and counted, then seeded at 75% confluence 24 hrs prior to transfection. Immediately prior to transfection, the cell media was replaced with fresh Opti-MEM. For lipoplex formation, pDNA at 60 µg/mL was slowly added to Lipofectamine 2000™ at a ratio of 1:2 and final nucleic acid concentration of 30 µg/mL in Opti-MEM. Lipoplexes were allowed to form at RT for 30 min. For soluble lipoplex delivery, the lipoplexes were added directly to the cell culture media. For MCM-mediated delivery, the lipoplexes were added to sterile MCMs at a ratio of 13.3 µg MCM:1 µg lipoplex (by nucleic acid mass). The MCM-lipoplex solution was incubated at RT and rotated for 30 min. The MCMs were then centrifuged at 2,000 g for 30 s, and the supernatant aspirated. The MCMs with bound lipoplexes were brought up in Opti-MEM and added directly to the cell culture media. The amount of bound lipoplexes was estimated to be 55% of the original 30 µg/mL, as indicated in the binding curve in S2C for 30 min of binding. Comparisons of transfection efficiency were assessed 36 hrs after transfection via epifluorescence microscopy (Nikon Ti Eclipse) for detection of EGFP+ cells (Fig. 2A,B). Efficiencies were compared at the stated concentrations (Fig. 2C) and reported as EGFP+ cells/cm^2^. After micrograph collection, the cells were assayed for metabolic activity. Briefly, a resazurin reduction assay, CellTiter-Blue (Promega), was used via direct addition of the dye to each culture well at a ratio of 20 µL dye:100 µL of culture media. The cells were incubated with the dye for 4 hrs and read for fluorescence at 590 nm/650 nm excitation and emission (Fluoroskan Ascent FL). The fluorescence signal for each well was normalized to control wells containing cells seeded at the same time and density as experimental groups and only treated with media exchange to OptiMEM at the time of transfection. Two-way ANOVA with Tukey post-hoc analysis was used to determine the statistical significance (p < 0.05) of MCMs on transfection efficiency and viability (GraphPad Prism). Transfections were carried out with three technical replicates. Relative trends in transfection were confirmed with an additional experimental replication.

### Scanning electron micrographs of hDF cultured with MCM

hDFs were cultured with 4.2 F MCMs on tissue culture-treated Thermanox Plastic Coverslips (Nunc) in DMEM with 10 v/v% FBS and 1000 U/mL P/S for 2 days. Cells were fixed on coverslips with 4% paraformaldehyde for 10 min at RT. Cells were further fixed in 0.7 M sodium cacodylate trihydrate (Sigma Aldrich) with 3 mM magnesium chloride (Sigma Aldrich) and 1.5 v/v% glutaraldehyde (Sigma-Aldrich) in DI water for 2 hrs at RT. Fixed cells were washed in 0.7 M sodium cacodylate with 2.5 w/v% sucrose 2X. Washed cells were dehydrated in 10 min subsequent incubations in 30, 50, 70, 95% ethanol in DI water. Dehydration was completed in 10 min subsequent incubations in 30, 50, 70, 95% hexamethyldisilazne (Sigma-Aldrich) in ethanol. Dehydrated cells were imaged on a LEO 1530 scanning electron microscope (Gemini) at 3kv. Cells and MCMs were pseudo colored in Adobe Photoshop to improve clarity of MCM and cellular regions.

### Characterization of MCMs’ localized effect

Human embryonic kidney cells (HEK293s) (ATCC) and primary hDFs were cultured in DMEM with 10 v/v% FBS and 1000 U/mL P/S. Lipoplexes were generated and adsorbed onto MCMs as described in methods 5.3. For colocalization effects (Fig. 3A), MCMs pre-labeled with a rhodamine-conjugated calcium phosphate-binding peptide were used to fluorescently identify the MCMs as previously described^49^. HEK293 were seeded so that approximately 50% of the cells were in contact with a red-labeled microparticle (S6). The cells were transfected as described in methods 5.3 and assessed for EGFP+ cells after 36 hrs. For analysis, a region of interest (ROI) that contained each EGFP+ cell was drawn in Nikon NIS-Elements. The ROIs were then evaluated as to whether they contained a red fluorescent MCM. Two-way ANOVA with Tukey post-hoc analysis was performed in GraphPad Prism to determine the statistical significance (p < 0.05) against a random association of 50%. To assess lipoplex release from MCMs (Fig. 4C), soluble lipoplexes or lipoplexes adsorbed onto MCMs were loaded into the upper left well of an iBidi µ-Slide 2 Well Co-Culture slide. After settling for 30 min at RT, HEK293s were seeded at approximately 90% confluence into the macrowells. Diffusion of viable lipoplexes within the soluble- or MCM-mediated lipoplex macrowells was assessed by EGFP+ cells via epifluorescence microscopy 36 hrs after transfection. For endosome analysis, hDFs were cultured with varying doses of 4.2 F MCMs (S9) or transfected with 4.2 F HA MCMs and rhodamine-labeled pEGFP as described in above. 1 hour after treatment or transfection, the cells were treated with 100 µg/mL pHrodo Green Dextran beads (ThermoFisher) for 10 min in 1XPBS. The staining solution was replaced with media and the cells were examined via time-lapse epifluorescence microscopy for 5 hrs in 10 minute intervals. All transfections were carried out with three technical replicates. Relative trends in transfection were confirmed with an additional experimental replication.

### Characterization of mineral coating dependence

5 µM diameter magnetic polystyrene beads (Spherotech) and 2–8 µm magnetite (iron (II) oxide) (Sigma-Aldrich) were coated and characterized in the same manner as described in methods 5.1. Transfection with the coated and uncoated materials was performed as described in methods 5.3. Two-way ANOVA with Tukey post-hoc analysis was used to determine the statistical significance (p < 0.05) of differences between the coated and uncoated materials (GraphPad Prism). Transfections were carried out with three technical replicates. Relative trends in transfection were confirmed with an additional experimental replication.

### Reverse transfection of H1 human embryonic stem cells

H1 human embryonic stem cells (hESC) (WiCell) were cultured on Matrigel-coated tissue culture polystyrene in Essential 8 (E8) (Life Technologies). Lipoplexes were formed in similar manner as described in methods 5.3, however E8 was used in all steps in place of OptiMEM. A polydimethylsiloxane (PDMS) stencil was used to create microwells on a glass petri dish. The PDMS microwells enabled the use of low volumes of lipoplex (MCM+/−) solutions to reduce large differences in lipoplex concentration between the soluble lipoplex and MCM-mediated reverse transfection methods, as well as isolation of small number of colonies for individual analysis and comparison. Soluble or lipoplexes bound to MCMs were added to the microwells and allowed to settle for 15 min. H1 hESCs were singularized using TrypLE (Thermo Fisher) and seeded at 10,000 cells per well. The wells were assessed for EGFP+ expression 36 hrs after transfection via epifluorescence microscopy (Nikon Ti Eclipse) (Fig. 2A). Transfections were carried out with three technical replicates. Relative trends in transfection were confirmed with an additional experimental replication. The percentage of EGFP+ colony and EGFP+ intensity over background signal was quantified using Nikon NIS-Elements. Oct4 and Nanog expression was assessed using immunocytochemistry and epifluorescence microscopy after 48 hrs of culture with MCMs. Briefly, cells were fixed for 15 min at RT in neutral buffered formalin and washed 3X with phosphate buffered saline (PBS). The cells were permeabilized for 30 min in 0.1% Triton X-100 (Sigma Aldrich)/PBS and then washed 3X in 0.05 v/v% Tween20 (Sigma Aldrich)/PBS. The cells were incubated with primary antibodies, rabbit anti-Nanog (Cell Signaling Technology 4903S 1:300 dilution) and mouse anti-Oct4 (Santa Cruz Biotechnology SC-5279 1:300 dilution) in 0.05 v/v% Tween20 for 30 min, and followed by three 5 minute washes in 0.05 v/v% Tween20. The cells were then incubated in Alexa Fluor 568-anti rabbit (Life Technologies 1:600 dilution), Alexa Fluor 488-anti mouse (Life Technologies 1:600 dilution), and DAPI (Life Technologies 1:1000 dilution) overnight at 4 °C. The stained cells were washed 3X in PBS and assessed for expression via epifluorescence microscopy (S4). The expression percentage was quantified in NIS-Elements. Briefly, a threshold and object count was used to define nuclei within each sample using the DAPI stain. The expression of green and red fluorescence for each nucleus was then measured and compared to background fluorescence. The percentage of nuclei positive above background cellular fluorescence for green or red fluorescence (Oct4/Nanog) is reported as the percentage of detected DAPI-positive objects.

### Comparison of 3-D transfection efficiency

For generation of cell aggregates, we utilized 400 µm agarose microwells formed from silicone molds as described in Dahlman *et al*.^50^. Briefly, 1.5 wt% molten agarose was added to sterile silicone molds and allowed to cool. A 6 mm biopsy punch was used to punch a disk of the formed microwells that fit a 96-well plate. The disks were then transferred to the well plate and centrifuged to the bottom of the well in media. The varied mineral coatings were formed as described in method 2.1 with the individual formulations detailed in S1. The lipoplex formation and adsorption to MCMs was performed as described in method 2.4. The HEK293s, hMSCs, and hDFs were singularized in 0.05 wt% trypsin/0.1 mM EDTA. For H1 hESCs, the cells were singularized in TrypLE after a 2 hour pre-treatment with 10 µM ROCK inhibitor Y-27632 (CalBiochem). The singularized cells were counted and then mixed with equal amounts of soluble lipoplexes or lipoplexes adsorbed to MCMs. The mixed solutions were then added to the well plate containing the agarose microwells, and centrifuged at 300 g for 5 min. Transgene expression was assessed at 36 hrs post-transfection via epifluorescence microscopy. Expression was quantified for 10 aggregates of each condition in triplicate as mean EGFP intensity above background (Nikon Elements). Two-way ANOVA with Tukey post-hoc analysis was used to determine the statistical significance (p < 0.05) of differences in EGFP expression (GraphPad Prism). Transfections were carried out with three technical replicates. Relative trends in transfection were confirmed with an additional experimental replication.

## Electronic supplementary material

Supplementary Information
