# Supplementary material for: Functionalization of microparticles with mineral coatings enhances non-viral transfection of primary human cells
Source: Sci Rep. 2017 Oct 27;7:14211. doi: 10.1038/s41598-017-14153-x (PMC5660152; doi:10.1038/s41598-017-14153-x)
Supplement: Supplementary file 1 — Supplementary Information [file 41598_2017_14153_MOESM1_ESM.doc]

**Functionalization of microparticles with mineral coatings enhances non-viral transfection of primary human cells.**

Andrew S. Khalil^1^, Xiaohua Yu^1^, Angela W. Xie^1^, Gianluca Fontana^1^, Jennifer M. Umhoefer^2^, Hunter J. Johnson^1^, Tracy A. Hookway^5,6^, Todd C. McDevitt^5,6^, William L. Murphy^1,2,3,4,*^

Supplemental Figures

| Reagent | MCM-A | | MCM-B | | MCM-C | | MCM-D | | MCM-E | | MCM-A* | | MCM-E* |
| --- | --- | --- | --- | --- | --- | --- | --- | --- | --- | --- | --- | --- | --- |
| **[NaCl] (mM)** | 141 | | | | | | | | | | | | |
| **[KCl] (mM)** | 4 | | | | | | | | | | | | |
| **[MgSO_4_●7H_2_O] (mM)** | 0.5 | | | | | | | | | | | | |
| **[MgCl_2_●6H_2_O] (mM)** | 1 | | | | | | | | | | | | |
| **[NaHCO_3_] (mM)** | *4.2* | *25* | | *50* | | *75* | | *100* | | *4.2* | | *100* | |
| **[HEPES] (mM)** | 20 | | | | | | | | | | | | |
| **[CaCl_2_●2H_2_O] (mM)** | 5 | | | | | | | | | | | | |
| **[KH_2_PO_4_] (mM)** | 2 | | | | | | | | | | | | |
| **[NaF] (mM)** | *0* | | | | | | | | | *1* | | | |

Supplement 1: mSBF formulations. MCM-Formulations listed in Fig. 6 for fluoride containing and fluoride-free formulations. Formulations were made daily by dissolving the listed reagents (in the order listed) to distilled water while stirring and heating to 37˚C. The mSBF was changed via centrifugation of the microparticles, decanting of the old mSBF, and replacement with freshly made solutions. This was performed for 5 days in 50mL conical tubes.


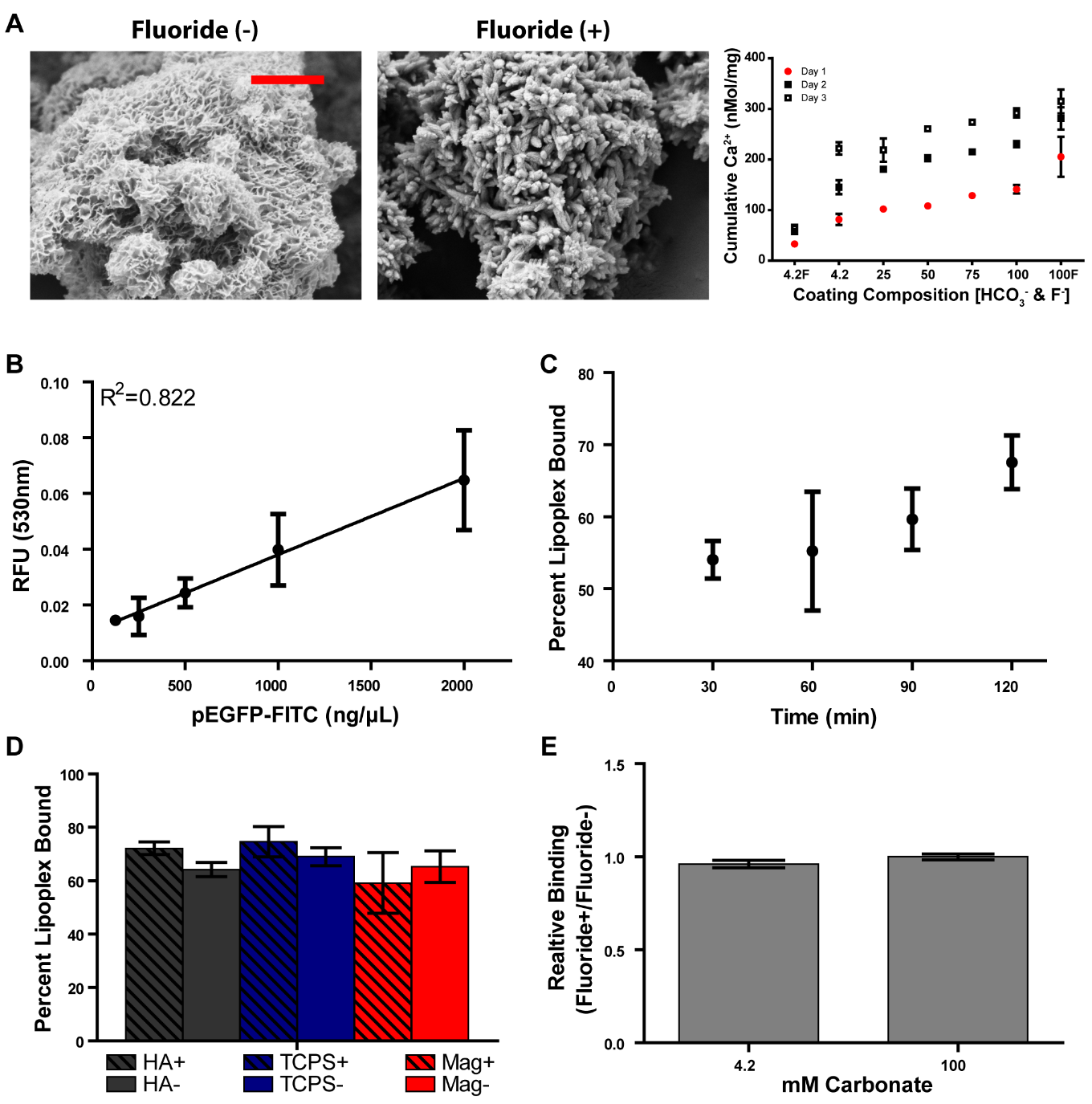


Supplement 2: Microparticle coating characterization. (A) Representative scanning electron micrographs of mineral coatings formed on microparticles. (left) Plate-like mineral coating morphology (100mM carbonate). (middle) “Needle-like” mineral coating morphology (100mM carbonate with 100mM fluoride). (right) Calcium release over transfection timeframe (3 days) from different carbonate and fluoride concentration mineral coatings. (B) Linearity of lipoplex standard curve using FITC-conjugated pDNA. (C) Lipoplex binding-over-time curve for 4.2F MCMs as measured by fluorescence depletion from binding solution. (D) Lipoplex binding of hydroxyapatite (HA), tissue culture polystyrene (TCPS), and magnetite (Mag) microparticles before and after mineralization. (E) Influence of fluoride-doping in mineral coating on lipoplex binding, as measured as ratio of bound lipoplex (Fluoride+:Fluoride-) in a fluorescence depletion assay. Scale bar = 1 µM

Supplement 3: Influence of MCM size on transfection efficiency. hDF were transfected with varying concentrations of pmCherry and examined for red fluorescence after 36 hrs using 4.2F-coated TCPS microspheres of 2, 6, and 16 µm.


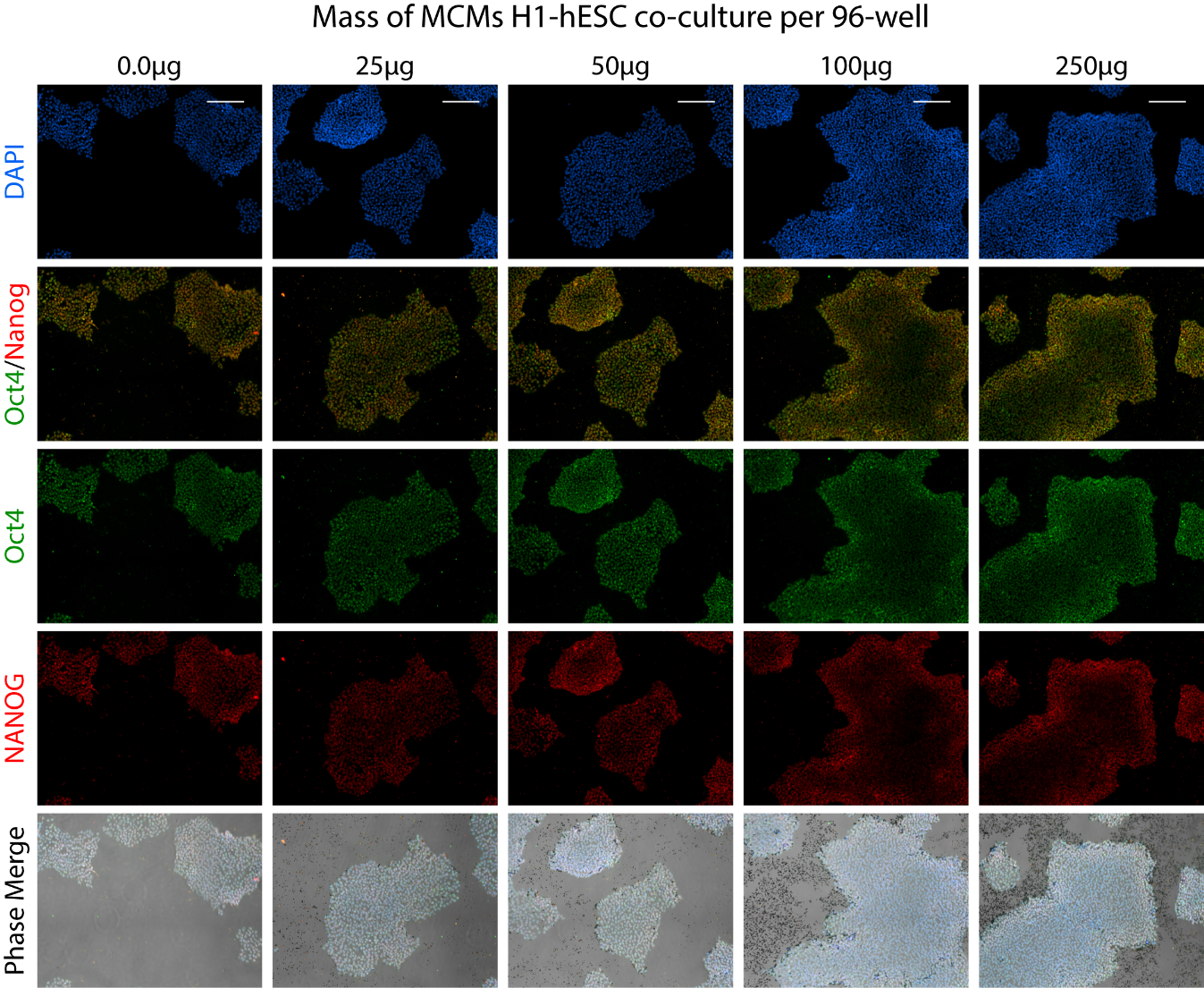


Supplement 4: MCMs influence on expression of pluripotency-associated transcription factor expression. Representative epifluorescence and phase micrographs of MCM-hESC co-culture for Oct4 (Alexfluor488) and Nanog (Alexfluor568) immunocytochemistry staining. H1-hESC were co-cultured with increasing MCM concentrations (µg/96-well) on Matrigel in E8 media for 48hrs. The MCM concentrations used ranged from 62.5% (25µg/96-well) to 625% (250µg/96-well) of the highest concentrations used in this study (40µg/96-well). No statistically significant differences were observed in percentages of Oct4+ or Nanog+ nuclei and no trend between MCM concentration and Oct4 or Nanog nuclear intensity could be established, indicating that MCM presence within these concentration ranges did not result in loss of expression of these pluripotency-associated transcription factors within the transfection-relevant timeframe of 48hrs. Scale bar = 250 µM

Supplement 5: Transfection reagent screen. We tested the MCM method with several commercial and previously established transfection reagents for increases in transfection efficiency (left) and cell viability (right). pmCherry-reagent complexation was carried out according to manufacturer’s recommendations or previously published standard methods for each reagent. *two-way ANOVA p –value < 0.05, ***two-way ANOVA p-value < 0.005


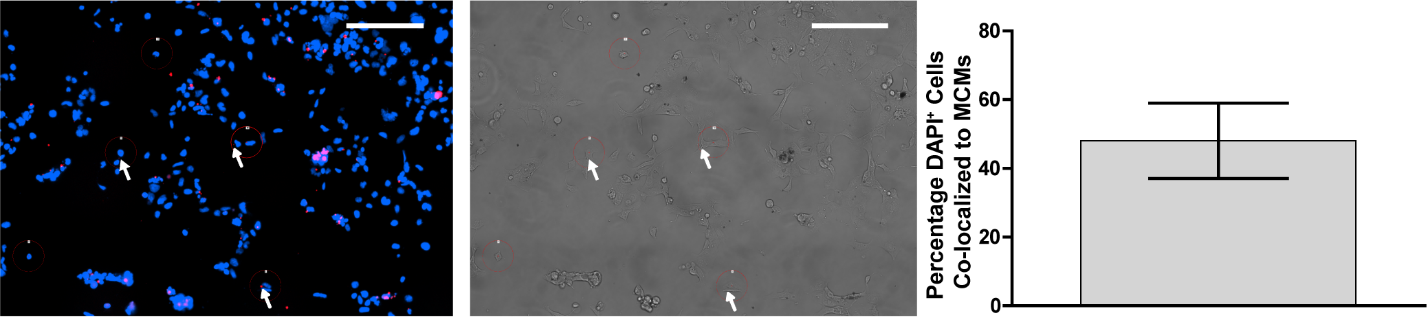


Supplement 6: Microparticle seeding density for HEK293 colocalization assay. (left) DAPI/rhodamine merged epifluorescence micrograph illustrating ROI determination centered on DAPI+ nucleic (arrow indicating ROI’s positive for both nuclei and rhodamine-labeled MCMs. (middle) Phase micrograph of ROIs demonstrating cellular cytoplasm size relationship to ROI (arrows indicating ROI’s containing a MCM). (right) DAPI+ cell-MCM association rate, determined as the rhodamine-labeled MCM+ ROI’s to the rhodamine-labeled MCM- ROIs for 10 DAPI+ centered ROI’s in 5 distinct wells. Scale bar = 50 µm.


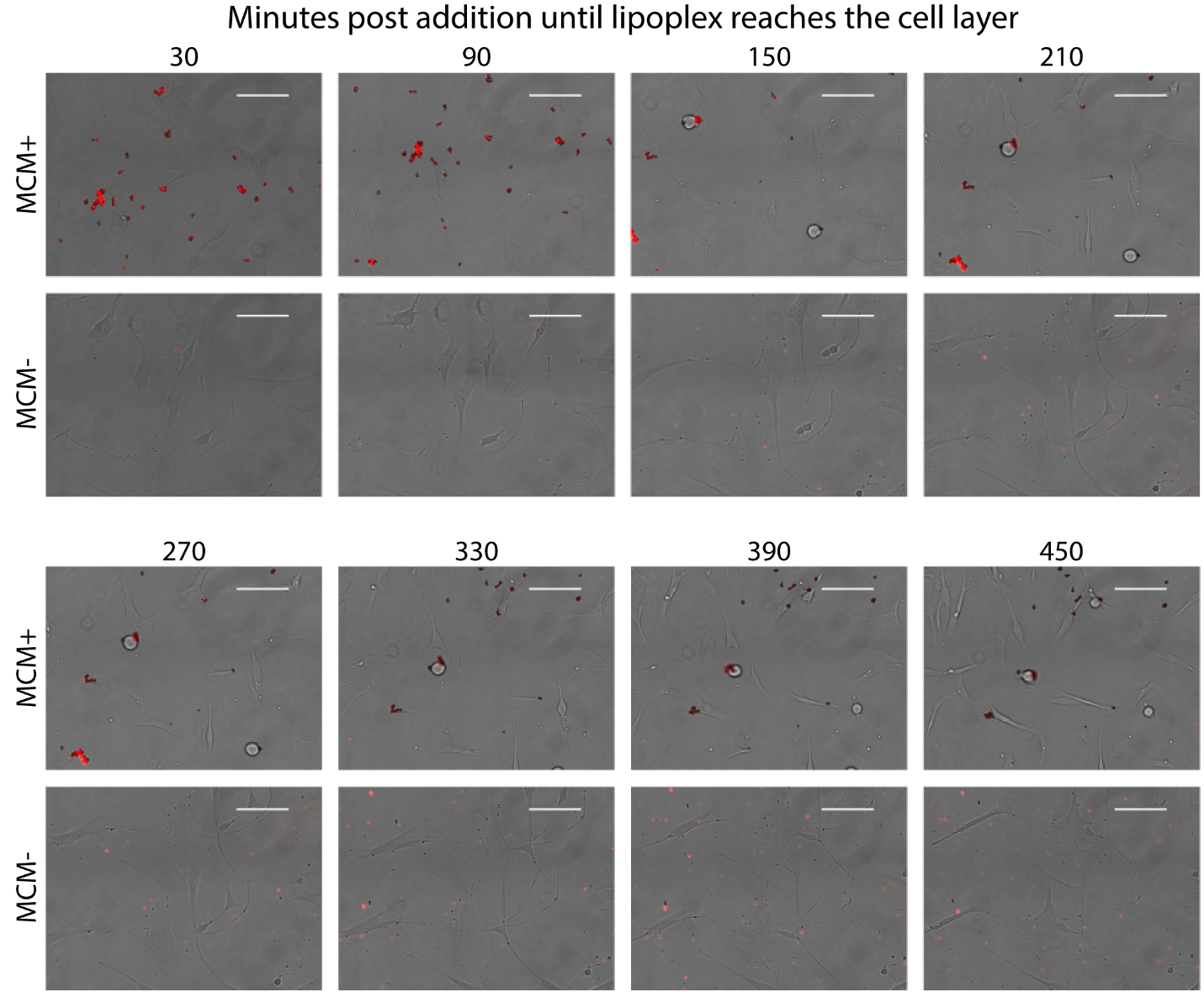


Supplement 7: Time-lapse microscopy of rhodamine-labeled pEGFP after addition to cell culture wells with and without MCMs. Time-lapse after addition (MCM+/-) in 30 minute increments. Rhodamine-labeled lipoplexes bound to MCMs (MCM+) were observed in the same focal plane as the cell layer in less than 5 minutes after addition where soluble rhodamine-labeled lipoplexes did not appear in the cell layer focal plane until approximately 450 minutes after addition. Scale bar = 100 µm


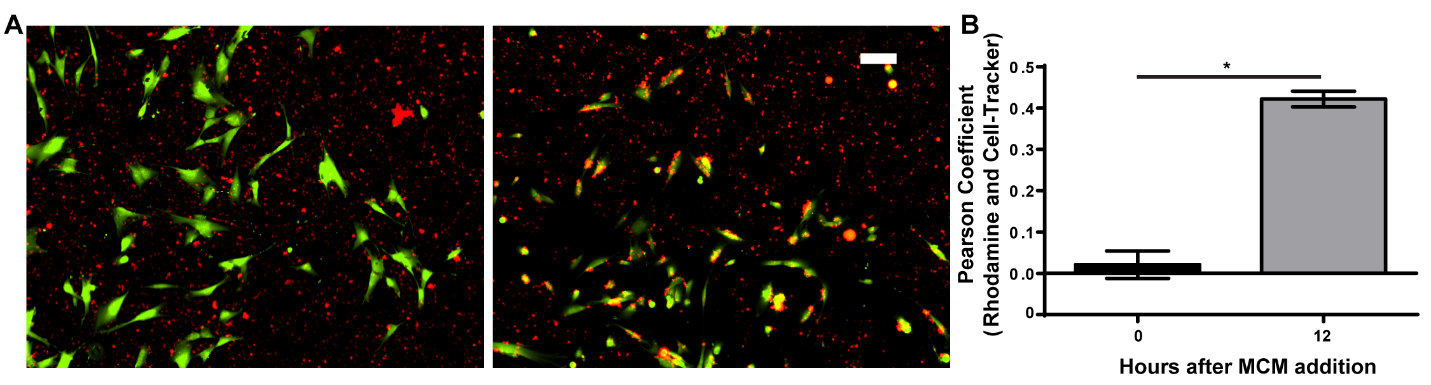


Supplement 8: MCM affinity for cells in 2-D culture. (A) Representative epifluorescence micrographs 0hr (left) and 12hr (right) after MCM addition to hDFs culture in 2-D. hDFs are labeled green with Cell Tracker Green (Life Technologies) and MCMs are labeled red with 5-carboxytetramethylrhodamine (Sigma-Aldrich)-labeled hydroxyapatite-binding peptide. (B) Pearson colocalization coefficient of rhodamine (MCMs) to Cell Tracker Green (cells) 0 and 12hr after MCM addition. Scale bar = 100 µm. p-value < 0.05.


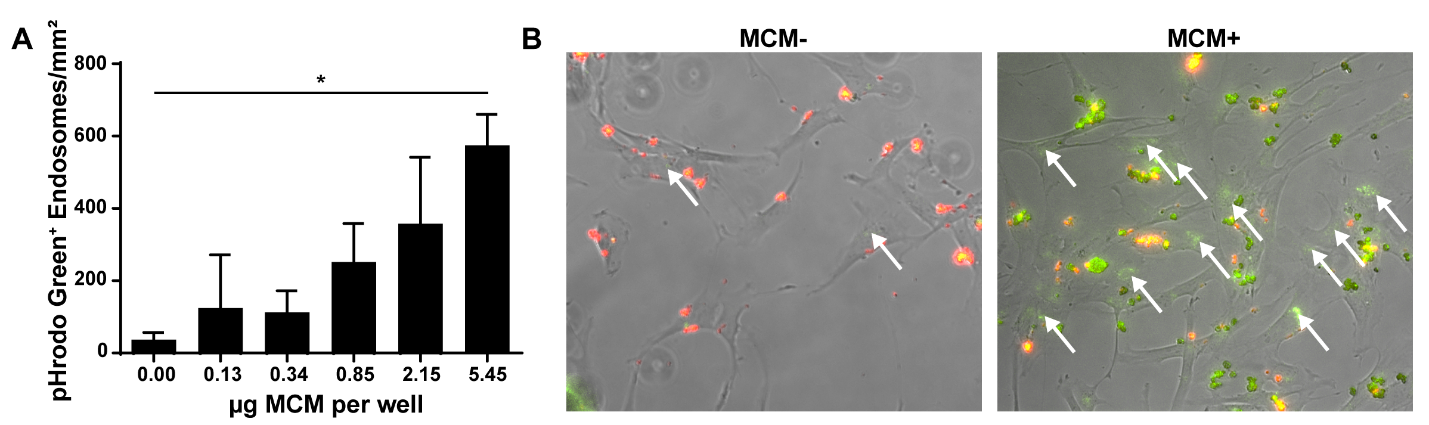


Supplement 9: hDFs cultured directly with 4.2F MCMs produce more endosomes than cells culture without. (A) hDFs were cultured with varying amounts of 4.2F MCMs per culture well for 1 hr prior to treatment with pHrodo Green-Dextran beads and subsequent assessment of endosome production via green fluorescence. *One-way ANOVA with post-test for linear trend p-value < 0.0001 (B) Merged phase and epifluorescence micrographs of hDFs transfected with rhodamine-labeled pEGFP-lipoplexes (+/-MCMs) and pHrodo Green-Dextran beads. Arrows indicate areas of cytoplasmic staining of pHrodo Green-Dextran.


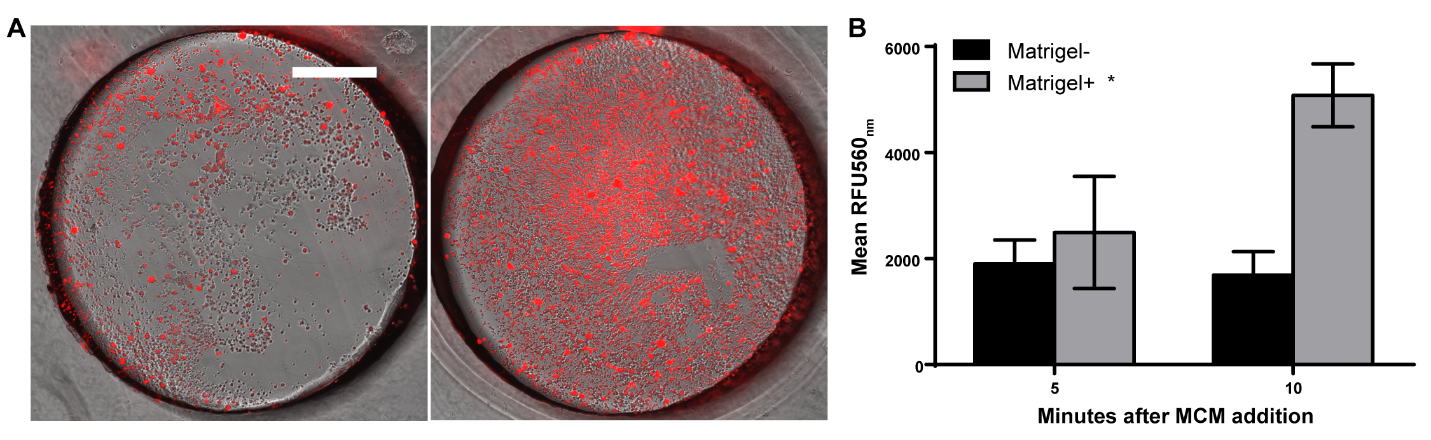


Supplement 10: MCMs rapidly adsorb to protein-coated substrates for reverse transfection. (A) Rhodamine-labeled MCMs were incubated in PBS for 5 and 10 minutes in 2.5mm PDMS microwells mounted on either glass (left) or Matrigel-coated glass (right). (B) Mean relative fluorescence intensity (640nm) for microwell area at 5 and 10 minutes. Scale bar = 500µm. *p-value < 0.05.

**
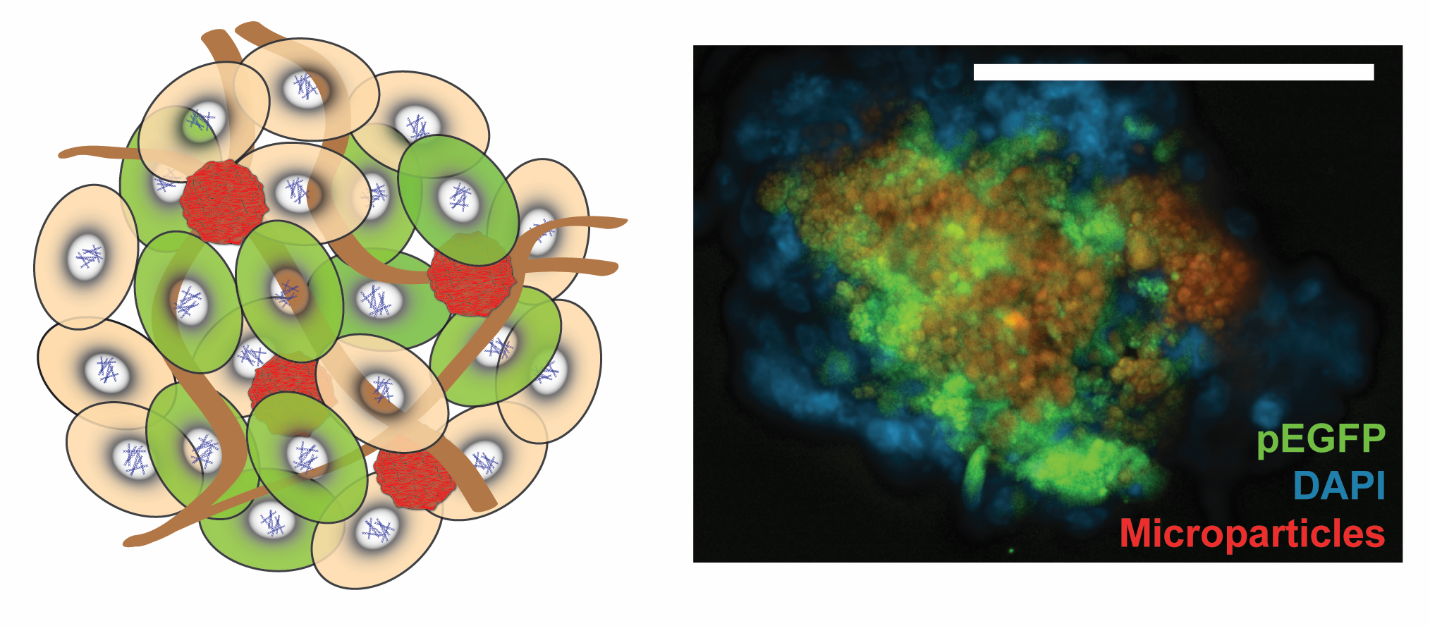
**Supplement 11: Confocal micrograph of MCM-transfected hDF aggregate. (left) Schematic illustrating MCM-mediated transfection of cell aggregates with lipoplex-laden MCMs and resulting EGFP+ cells throughout the aggregate interior. (right) Confocal micrograph showing a 5µm optical slice of transfected hDF aggregate with rhodamine-labeled MCMs in red, transfected EGFP+ cells in green, and DAPI+ nuclei in blue. Optical slice is 50µm into the interior of the aggregate. Scale bar = 50µm.
